# Supplementary material for: The Role of Muscle Mass Gain Following Protein Supplementation Plus Exercise Therapy in Older Adults with Sarcopenia and Frailty Risks: A Systematic Review and Meta-Regression Analysis of Randomized Trials
Source: Nutrients. 2019 Jul 25;11(8):1713. doi: 10.3390/nu11081713 (PMC6723070; doi:10.3390/nu11081713)
Supplement: Supplementary file 1 [file nutrients-11-01713-s001.pdf]

Table S1. Database search formulas

Data base	Search terms for query
**Pubmed**	
#1	((((frailty/) OR frail) OR sarcopenia) OR elderly) OR order adults
#2	[(nursing home) OR (institution)] residents
#3	(((progressive resistance training) OR resistance exercise) OR strength training) OR strengthening exercise
#4	(((weight training) OR weight lifting/) OR weighted exercise
#5	multicomponent exercise
#6	(physical activity exercise) OR function training
#7	[(whey protein) Or (amino-acid) OR (leucine)] supplement
#8	[(diet) OR (nutrient)] intervention
#9	[(nutrient) OR (nutrition)] supplement
#10	(#1) OR 2
#11	(((#3) OR #4) OR #5) OR #6
#12	((#7) OR #8) OR #9
#13	((#10) AND #11) AND #12 AND (randomized controlled trial)
**Physiotherapy Evidence Database (PEDro)**	
	Method: clinical trial
	Abstract & Title:
#1	frail elderly
#2	frailty
#3	sarcopenia
#4	resistance training
#5	strengthening exercise
#6	multicomponent exercise
#7	physical activity exercise
#8	protein supplement
#9	whey protein supplement

(continued)

Table S1. (continued)

Data base	Search terms for query
**Excerpta Medica dataBASE (EMBASE)**	
#1	sarcopenia
#2	frailty
#3	frail
#4	elderly
#5	Older adults
#6	('nursing'/exp OR nursing) AND ('home'/exp OR home)
#7	'institutional care'
#8	#1 OR #2 OR #3 OR #4 OR #5 OR #6 OR #7
#9	Resistance AND training OR exercise
#10	strength AND training OR exercise
#11	strengthening exercise
#12	multicomponent AND exercise
#13	physical AND activity AND exercise AND training
#14	function training
#15	#9 OR #10 OR #11 OR #12 OR #13 OR #14
#16	whey protein supplement
#17	whey protein
#18	leucine
#19	nutrition AND supplement
#20	nutrient AND supplement
#21	#16 OR #17 OR #18 OR #19 OR #20
#22	#8 AND #15 AND #21
#23	#22 AND [randomized controlled trial]/lim AND ([article]/lim OR [article in press]/lim) AND [humans]/lim
**Cochrane Library Database**	
#1	frailty
#2	sarcopenia
#3	resistance training
#4	strengthening exercise
#5	multicomponent exercise
#6	physical activity exercise
#7	protein supplement
#8	#1 OR #2
#9	#3 OR #4 OR #5 OR #6
#10	#7 AND #8 AND #9

(continued)

Table S1. (continued)

Data base	Search terms for query
**China knowledge resource integrated database**	
#1	(frailty) OR (frail elderly)
#2	exercise training
#3	(whey protein) OR (leucine)
#4	#1 AND #2 AND #3 AND (randomized controlled trial)
**Google Scholar**	
#1	allintitle: frail elderly OR frailty
#2	allintitle: resistance training OR strengthening exercise
#3	allintitle: multicomponent exercise OR physical activity exercise
#4	allintitle: protein supplement OR whey OR leucine

Figure S1

Study or Subgroup	PS plus MSE Mean	PS plus MSE SD	PS plus MSE Total	Control Mean	Control SD	Control Total	Weight	Std. Mean Difference IV, Random, 95% CI
**LBM, overall, random model**								
Bjorkman 2011, RET+PS vs RET+PLA-S	0.4	2.45	40	0.2	4.53	40	4.1%	0.05 [-0.38, 0.49]
Bonnefoy 2003, MET+PS vs PLA-S	0.6	1.39	20	-0.35	1.14	20	3.6%	0.73 [0.09, 1.37]
Bonnefoy 2012, MET+PS vs Con	0.84	8.5	53	-1.4	6.5	49	4.2%	0.29 [-0.10, 0.68]
Carlsson 2011, MET+PS vs CG1 MET+PLA-S	-0.1	2.37	13	-0.3	2.13	26	3.5%	0.09 [-0.58, 0.75]
Carlsson 2011, MET+PS vs CG2 PS	0.01	1.84	12	-0.6	3.46	40	3.6%	0.19 [-0.46, 0.84]
Carlsson 2011, MET+PS vs CG3 PLA-S	-0.1	2.37	12	0	2.36	39	3.6%	-0.04 [-0.69, 0.61]
Dirks 2017, RET+PS vs RET+PLA-S	1.4	2.3	17	-0.1	1.97	17	3.5%	0.68 [-0.01, 1.38]
Englund 2017, MET+PS vs MET+PLA-S	0.13	1.54	60	0.45	1.66	57	4.3%	-0.20 [-0.56, 0.16]
Fiatarone 1994, RET+PS vs CG1 RET+PLA-S	1.5	3.91	9	0.4	4.61	25	3.3%	0.24 [-0.52, 1.01]
Fiatarone 1994, RET+PS vs CG2 PS	1.5	3.91	8	1	3.73	24	3.2%	0.13 [-0.67, 0.93]
Fiatarone 1994, RET+PS vs CG3 PLA-S	1.5	3.91	8	0.2	4.39	26	3.2%	0.30 [-0.50, 1.09]
Hegerova 2015, MET+PS vs Con	-1	1.86	100	-4.9	3.37	100	4.4%	1.43 [1.12, 1.74]
Imaoka 2016, MET+PS vs CG1 MET	0.44	0.27	8	-0.29	0.2	22	2.3%	3.23 [2.04, 4.43]
Imaoka 2016, MET+PS vs CG2 PS	0.44	0.27	8	-0.19	0.27	23	2.7%	2.27 [1.27, 3.28]
Imaoka 2016, MET+PS vs CG3 Con	0.44	0.27	7	-0.39	0.27	23	2.3%	2.99 [1.82, 4.16]
Kim 2012, MET+PS vs CG1 MET	0.6	0.64	12	0.4	0.65	36	3.6%	0.30 [-0.35, 0.96]
Kim 2012, MET+PS vs CG2 PS	0.6	0.64	11	0.2	0.49	37	3.5%	0.75 [0.06, 1.44]
Kim 2012, MET+PS vs CG3 Con	0.6	0.64	11	0.1	0.47	37	3.5%	0.96 [0.26, 1.66]
Kim 2016, MET+PS vs CG1 MET	1.1	3	12	0.1	4.14	35	3.6%	0.25 [-0.41, 0.91]
Kim 2016, MET+PS vs CG2 PS	1.1	3	12	0.6	3.49	34	3.6%	0.15 [-0.51, 0.80]
Kim 2016, MET+PS vs CG3 Con	1.1	3	12	1.2	4.67	34	3.6%	-0.02 [-0.68, 0.64]
Maltais 2016, RET+EAA-PS vs RET+PLA-S	2.29	1.13	8	1.62	1.03	5	2.4%	0.57 [-0.58, 1.72]
Maltais 2016, RET+Milk-PS vs RET+PLA-S	2.17	1.58	8	1.62	1.03	5	2.4%	0.36 [-0.77, 1.49]
Molnar 2016, MET+PS vs MET	0.5	2.2	17	-1.05	1.1	17	3.4%	0.87 [0.16, 1.58]
Rondanelli 2016, MET+PS vs PLA-S	1.38	2.22	69	-0.32	2.41	61	4.3%	0.73 [0.38, 1.09]
Tieland 2012, RET+PS vs RET+PLA-S	1.2	1.3	28	-0.1	1.3	28	3.8%	0.99 [0.43, 1.54]
Yamada 2015 RET+PS vs CG1 RET	0.2	0.2	16	0.1	0.3	15	3.4%	0.38 [-0.33, 1.10]
Yamada 2015 RET+PS vs CG2 Con	0.2	0.2	15	-0.3	0.28	25	3.2%	1.93 [1.15, 2.71]
Zdzieblik 2015, RET+PS vs RET+PLA-S	4.2	2.3	26	2.9	1.8	27	3.8%	0.62 [0.07, 1.17]
**Subtotal (95% CI)**			**632**			**927**	**100.0%**	**0.66 [0.41, 0.91]**
Heterogeneity: Tau² = 0.35; Chi² = 132.84, df = 28 (P < 0.00001); I² = 79%								
Test for overall effect: Z = 5.14 (P < 0.00001)								
**Total lean mass, ≥6 mo, <12 mo, random model**								
Bonnefoy 2003, MET+PS vs PLA-S	0.3	1.39	20	-0.45	1.14	20	9.9%	0.58 [-0.06, 1.21]
Carlsson 2011, MET+PS vs CG1 MET+PLA-S	-0.1	2.37	13	-0.3	2.13	26	9.7%	0.09 [-0.58, 0.75]
Carlsson 2011, MET+PS vs CG2 PS	-0.1	2.37	12	-0.6	4.4	37	9.8%	0.12 [-0.53, 0.77]
Carlsson 2011, MET+PS vs CG3 PLA-S	-0.1	2.37	12	0	2.36	39	9.8%	-0.04 [-0.69, 0.61]
Dirks 2017, RET+PS vs RET+PLA-S	1.4	2.3	17	-0.1	1.97	17	9.5%	0.68 [-0.01, 1.38]
Englund 2017, MET+PS vs MET+PLA-S	0.13	1.54	60	0.45	1.66	57	11.2%	-0.20 [-0.56, 0.16]
Hegerova 2015, MET+PS vs Con	-1	1.86	100	-4.9	3.37	100	11.4%	1.43 [1.12, 1.74]
Tieland 2012, RET+PS vs RET+PLA-S	1.25	2.7	28	-0.4	1.83	28	10.4%	0.71 [0.16, 1.25]
Yamada 2015 RET+PS vs CG1 RET	0.2	0.2	16	0.1	0.3	15	9.4%	0.38 [-0.33, 1.10]
Yamada 2015 RET+PS vs CG2 Con	0.2	0.2	15	-0.3	0.28	25	9.0%	1.93 [1.15, 2.71]
**Subtotal (95% CI)**			**293**			**364**	**100.0%**	**0.56 [0.09, 1.03]**
Heterogeneity: Tau² = 0.48; Chi² = 65.52, df = 9 (P < 0.00001); I² = 86%								
Test for overall effect: Z = 2.35 (P = 0.02)								
**Total lean mass, ≥3 mo, <6 mo, random model**								
Bonnefoy 2003, MET+PS vs PLA-S	0.6	1.39	20	-0.35	1.14	20	4.8%	0.73 [0.09, 1.37]
Bonnefoy 2012, MET+PS vs Con	0.84	8.5	53	-1.4	6.5	49	6.0%	0.29 [-0.10, 0.68]
Carlsson 2011, MET+PS vs CG1 MET+PLA-S	0.01	1.84	12	-0.1	1.77	30	4.7%	0.06 [-0.61, 0.73]
Carlsson 2011, MET+PS vs CG2 PS	0.01	1.84	12	-0.6	3.46	40	4.8%	0.19 [-0.46, 0.84]
Carlsson 2011, MET+PS vs CG3 PLA-S	0.01	1.84	12	0.1	1.72	43	4.8%	-0.05 [-0.69, 0.59]
Dirks 2017, RET+PS vs RET+PLA-S	1.3	2.3	17	0.1	1.97	17	4.6%	0.55 [-0.14, 1.23]
Hegerova 2015, MET+PS vs Con	-0.4	1.81	100	-3.5	3.77	100	6.4%	1.04 [0.75, 1.34]
Imaoka 2016, MET+PS vs CG1 MET	0.44	0.27	8	-0.29	0.2	22	2.7%	3.23 [2.04, 4.43]
Imaoka 2016, MET+PS vs CG2 PS	0.44	0.27	8	-0.19	0.27	23	3.3%	2.27 [1.27, 3.28]
Imaoka 2016, MET+PS vs CG3 Con	0.44	0.27	7	-0.39	0.27	23	2.8%	2.99 [1.82, 4.16]
Kim 2012, MET+PS vs CG1 MET	0.6	0.64	12	0.4	0.65	36	4.8%	0.30 [-0.35, 0.96]
Kim 2012, MET+PS vs CG2 PS	0.6	0.64	11	0.2	0.49	37	4.6%	0.75 [0.06, 1.44]
Kim 2012, MET+PS vs CG3 Con	0.6	0.64	11	0.1	0.47	37	4.5%	0.96 [0.26, 1.66]
Kim 2016, MET+PS vs CG1 MET	1.1	3	12	0.1	4.14	35	4.7%	0.25 [-0.41, 0.91]
Kim 2016, MET+PS vs CG2 PS	1.1	3	12	0.6	3.49	34	4.7%	0.15 [-0.51, 0.80]
Kim 2016, MET+PS vs CG3 Con	1.1	3	12	1.2	4.67	34	4.7%	-0.02 [-0.68, 0.64]
Maltais 2016, RET+EAA-PS vs RET+PLA-S	2.29	1.13	8	1.62	1.03	5	2.9%	0.57 [-0.58, 1.72]
Maltais 2016, RET+Milk-PS vs RET+PLA-S	2.17	1.58	8	1.62	1.03	5	2.9%	0.36 [-0.77, 1.49]
Molnar 2016, MET+PS vs MET	0.5	2.2	17	-1.05	1.1	17	4.5%	0.87 [0.16, 1.58]
Rondanelli 2016, MET+PS vs PLA-S	1.38	2.22	69	-0.32	2.41	61	6.1%	0.73 [0.38, 1.09]
Tieland 2012, RET+PS vs RET+PLA-S	1.2	1.3	28	-0.1	1.3	28	5.2%	0.99 [0.43, 1.54]
Zdzieblik 2015, RET+PS vs RET+PLA-S	4.2	2.3	26	2.9	1.8	27	5.2%	0.62 [0.07, 1.17]
**Subtotal (95% CI)**			**475**			**723**	**100.0%**	**0.71 [0.45, 0.96]**
Heterogeneity: Tau² = 0.24; Chi² = 74.72, df = 21 (P < 0.00001); I² = 72%								
Test for overall effect: Z = 5.47 (P < 0.00001)								
**Total lean mass, <3 mo, random model**								
Bjorkman 2011, RET+PS vs RET+PLA-S	0.4	2.45	40	0.2	4.53	40	21.8%	0.05 [-0.38, 0.49]
Fiatarone 1994, RET+PS vs CG1 RET+PLA-S	1.5	3.91	9	0.4	4.61	25	18.7%	0.24 [-0.52, 1.01]
Fiatarone 1994, RET+PS vs CG2 PS	1.5	3.91	8	1	3.73	24	18.3%	0.13 [-0.67, 0.93]
Fiatarone 1994, RET+PS vs CG3 PLA-S	1.5	3.91	8	0.2	4.39	26	18.4%	0.30 [-0.50, 1.09]
Hegerova 2015, MET+PS vs Con	1.3	1.86	100	-3.9	4.8	100	22.8%	1.42 [1.11, 1.73]
**Subtotal (95% CI)**			**165**			**215**	**100.0%**	**0.46 [-0.26, 1.17]**
Heterogeneity: Tau² = 0.56; Chi² = 32.82, df = 4 (P < 0.00001); I² = 88%								
Test for overall effect: Z = 1.26 (P = 0.21)								

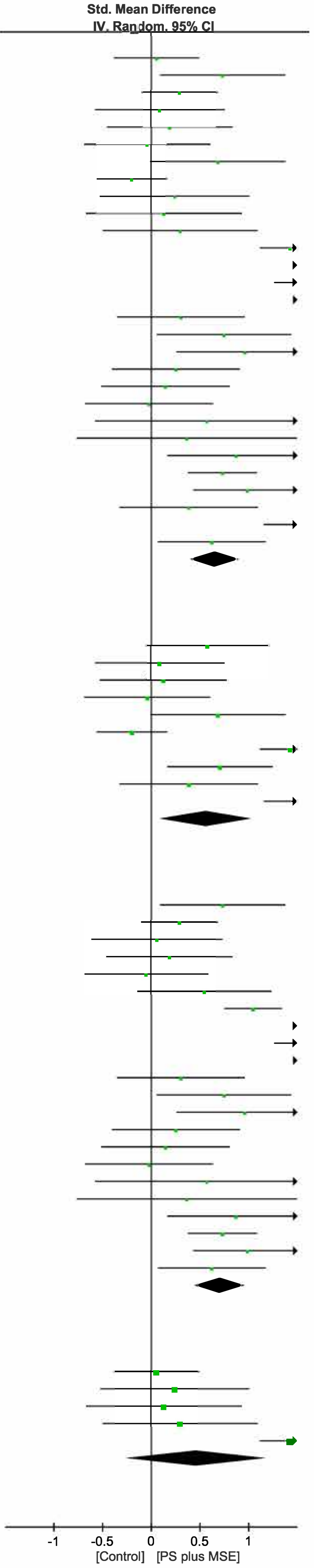

Figure S1. Forest plot summarizing effects of protein supplement (PS) plus muscle strength exercise training (MSE) on lean body mass at an overall duration and each follow-up time point. The horizontal line links the lower and upper limits of the 95% CI of this effect. The combined effects are plotted using black diamonds. 95% CI = 95% confidence interval; Std. = standard; IV = inverse variance; CG = control group; Con = control; MET = multicomponent exercise training; PLA-S, placebo supplement; RET = resistance exercise training.

Figure S2

Study or Subgroup	PS plus MSE Mean	SD	Total	Control Mean	SD	Total	Weight	Std. Mean Difference IV, Random, 95% CI
**Appendicular lean mass, overall, random model**								
Bjorkman 2011, RET+PS vs RET+PLA-S (1)	0.9	4.5	40	0.2	5.28	40	7.9%	0.14 [-0.30, 0.58]
Dirks 2017, RET+PS vs RET+PLA-S	1	1.22	17	0	0.95	17	5.7%	0.89 [0.18, 1.60]
Englund 2017, MET+PS vs MET+PLA-S	0.16	0.85	60	0.09	0.82	57	8.5%	0.08 [-0.28, 0.45]
Kim 2012, MET+PS vs CG1 MET	0.4	0.34	12	0.2	0.36	36	6.1%	0.55 [-0.11, 1.22]
Kim 2012, MET+PS vs CG2 PS	0.4	0.34	11	0.1	0.24	37	5.7%	1.11 [0.40, 1.83]
Kim 2012, MET+PS vs CG3 Con	0.4	0.27	11	0.03	0.19	37	5.4%	1.73 [0.97, 2.50]
Kim 2016, MET+PS vs CG1 MET	0.2	0.46	12	0.3	0.44	35	6.1%	-0.22 [-0.88, 0.44]
Kim 2016, MET+PS vs CG2 PS	0.2	0.46	12	0.2	0.48	34	6.1%	0.00 [-0.66, 0.66]
Kim 2016, MET+PS vs CG3 Con	0.2	0.46	12	0.3	0.33	34	6.1%	-0.27 [-0.93, 0.39]
Maltais 2016, RET+EAA-PS vs RET+PLA-S	0.45	0.32	8	0.44	0.34	5	3.5%	0.03 [-1.09, 1.15]
Maltais 2016, RET+Milk-PS vs RET+PLA-S	0.41	0.26	8	0.44	0.34	5	3.5%	-0.10 [-1.21, 1.02]
Rondanelli 2016, MET+PS vs PLA-S	0.21	0.58	69	-0.06	0.59	61	8.6%	0.46 [0.11, 0.81]
Tieland 2012, RET+PS vs RET+PLA-S	0.95	1.43	28	-0.2	1.91	28	7.0%	0.67 [0.13, 1.21]
Yamada 2019, dynapenia, RET+PS vs CG1 RET	-0.32	1.35	6	0.08	1.11	20	4.4%	-0.33 [-1.25, 0.58]
Yamada 2019, dynapenia, RET+PS vs CG2 PS	-0.32	1.35	6	-0.2	1.09	19	4.4%	-0.10 [-1.02, 0.82]
Yamada 2019, dynapenia, RET+PS vs CG3 Con	-0.32	1.35	7	-0.65	1.7	20	4.7%	0.20 [-0.67, 1.06]
Yamada 2019, sarcopenia, RET+PS vs CG1 RET	0.61	0.4	3	0.09	0.5	8	2.5%	0.99 [-0.43, 2.42]
Yamada 2019, sarcopenia, RET+PS vs CG2 PS	0.61	0.4	3	0.07	0.28	9	2.2%	1.62 [0.09, 3.15]
Yamada 2019, sarcopenia, RET+PS vs CG3 Con	0.61	0.4	3	-0.88	0.58	8	1.6%	2.50 [0.64, 4.36]
**Subtotal (95% CI)**			**328**			**510**	**100.0%**	**0.40 [0.15, 0.66]**
Heterogeneity: Tau² = 0.17; Chi² = 44.31, df = 18 (P = 0.0005); I² = 59%								
Test for overall effect: Z = 3.10 (P = 0.002)								
**Appendicular lean mass, ≥6 mo, <12 mo, fixed model**								
Dirks 2017, RET+PS vs RET+PLA-S	1	1.22	17	0	0.95	17	15.3%	0.89 [0.18, 1.60]
Englund 2017, MET+PS vs MET+PLA-S	0.16	0.85	60	0.09	0.82	57	58.4%	0.08 [-0.28, 0.45]
Tieland 2012, RET+PS vs RET+PLA-S	0.95	1.43	28	-0.2	1.91	28	26.4%	0.67 [0.13, 1.21]
**Subtotal (95% CI)**			**105**			**102**	**100.0%**	**0.36 [0.09, 0.64]**
Heterogeneity: Chi² = 5.69, df = 2 (P = 0.06); I² = 65%								
Test for overall effect: Z = 2.56 (P = 0.01)								
**Appendicular lean mass, ≥3 mo, <6 mo, random model** Dirks								
2017, RET+PS vs RET+PLA-S	0.4	1.07	17	0.1	0.95	17	7.1%	0.29 [-0.39, 0.97]
Kim 2012, MET+PS vs CG1 MET	0.4	0.34	12	0.2	0.36	36	7.2%	0.55 [-0.11, 1.22]
Kim 2012, MET+PS vs CG2 PS	0.4	0.34	11	0.1	0.24	37	6.8%	1.11 [0.40, 1.83]
Kim 2012, MET+PS vs CG3 Con	0.4	0.27	11	0.03	0.19	37	6.4%	1.73 [0.97, 2.50]
Kim 2016, MET+PS vs CG1 MET	0.2	0.46	12	0.3	0.44	35	7.2%	-0.22 [-0.88, 0.44]
Kim 2016, MET+PS vs CG2 PS	0.2	0.46	12	0.2	0.48	34	7.2%	0.00 [-0.66, 0.66]
Kim 2016, MET+PS vs CG3 Con	0.2	0.46	12	0.3	0.33	34	7.2%	-0.27 [-0.93, 0.39]
Maltais 2016, RET+EAA-PS vs RET+PLA-S	0.45	0.32	8	0.44	0.34	5	4.3%	0.03 [-1.09, 1.15]
Maltais 2016, RET+Milk-PS vs RET+PLA-S	0.41	0.26	8	0.44	0.34	5	4.3%	-0.10 [-1.21, 1.02]
Rondanelli 2016, MET+PS vs PLA-S	0.21	0.58	69	-0.06	0.59	61	9.8%	0.46 [0.11, 0.81]
Tieland 2012, RET+PS vs RET+PLA-S	0.4	0.64	28	0	2.61	28	8.3%	0.21 [-0.32, 0.73]
Yamada 2019, dynapenia, RET+PS vs CG1 RET	-0.32	1.35	6	0.08	1.11	20	5.4%	-0.33 [-1.25, 0.58]
Yamada 2019, dynapenia, RET+PS vs CG2 PS	-0.32	1.35	6	-0.2	1.09	19	5.3%	-0.10 [-1.02, 0.82]
Yamada 2019, dynapenia, RET+PS vs CG3 Con	-0.32	1.35	7	-0.65	1.7	20	5.7%	0.20 [-0.67, 1.06]
Yamada 2019, sarcopenia, RET+PS vs CG1 RET	0.61	0.4	3	0.09	0.5	8	3.1%	0.99 [-0.43, 2.42]
Yamada 2019, sarcopenia, RET+PS vs CG2 PS	0.61	0.4	3	0.07	0.28	9	2.8%	1.62 [0.09, 3.15]
Yamada 2019, sarcopenia, RET+PS vs CG3 Con	0.61	0.4	3	-0.88	0.58	8	2.0%	2.50 [0.64, 4.36]
**Subtotal (95% CI)**			**228**			**413**	**100.0%**	**0.38 [0.09, 0.68]**
Heterogeneity: Tau² = 0.19; Chi² = 38.04, df = 16 (P = 0.001); I² = 58%								
Test for overall effect: Z = 2.59 (P = 0.010)								
**Appendicular lean mass, <3 mo**								
Bjorkman 2011, RET+PS vs RET+PLA-S (2)	0.9	4.5	40	0.2	5.28	40	100.0%	0.14 [-0.30, 0.58]
**Subtotal (95% CI)**			**40**			**40**	**100.0%**	**0.14 [-0.30, 0.58]**
Heterogeneity: Not applicable								
Test for overall effect: Z = 0.63 (P = 0.53)								

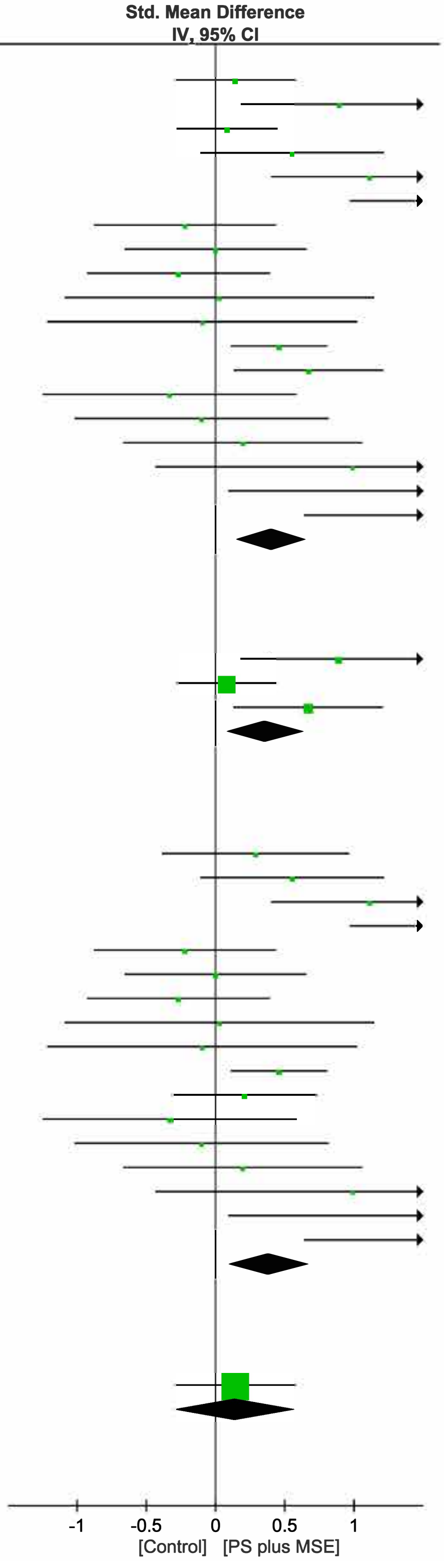

Figure S2. Forest plot summarizing effects of protein supplement (PS) plus muscle strength exercise training (MSE) on appendicular lean mass at an overall duration and each follow-up time point. The horizontal line links the lower and upper limits of the 95% CI of this effect. The combined effects are plotted using black diamonds. 95% CI = 95% confidence interval; Std. = standard; IV = inverse variance; CG = control group; Con = control; MET = multicomponent exercise training; PLA-S, placebo supplement; RET = resistance exercise training.

Figure S3

Study or Subgroup	PS plus MSE Mean	SD	Total	Control Mean	SD	Total	Weight	Std. Mean Difference IV, Fixed, 95% CI
**Handgrip strength, overall, fixed model**								
Bjorkman 2011, RET+PS vs RET+PLA-S	3.7	27.3	46	2.2	24.5	46	19.0%	0.06 [-0.35, 0.47]
Dirks 2017, RET+PS vs RET+PLA-S	3.5	6	17	0.5	7.4	17	6.8%	0.43 [-0.25, 1.12]
Kim 2016, MET+PS vs CG1 MET	0.4	3.2	12	-0.2	2.4	35	7.3%	0.23 [-0.43, 0.88]
Kim 2016, MET+PS vs CG2 PS	0.4	3.2	12	0.5	3.3	34	7.3%	-0.03 [-0.69, 0.63]
Kim 2016, MET+PS vs CG3 Con	0.4	3.2	12	-0.3	2.7	34	7.3%	0.24 [-0.42, 0.90]
Rondanelli 2016, MET+PS vs PLA-S	3.2	4	69	-0.47	2.3	61	23.1%	1.10 [0.73, 1.47]
Tieland 2012, RET+PS vs RET+PLA-S	2.2	4.6	31	0.45	4.6	31	12.6%	0.38 [-0.13, 0.88]
Yamada 2019, dynapenia, RET+PS vs CG1 RET	0.46	1.82	6	0.21	2.26	20	3.8%	0.11 [-0.80, 1.02]
Yamada 2019, dynapenia, RET+PS vs CG2 PS	0.46	1.82	6	0.04	3.63	19	3.8%	0.12 [-0.80, 1.04]
Yamada 2019, dynapenia, RET+PS vs CG3 Con	0.46	1.82	7	-0.14	1.94	20	4.2%	0.30 [-0.56, 1.17]
Yamada 2019, sarcopenia, RET+PS vs CG1 RET	1.37	1.69	3	-0.53	2.06	8	1.6%	0.88 [-0.53, 2.28]
Yamada 2019, sarcopenia, RET+PS vs CG2 PS	1.37	1.69	3	-0.46	1.65	9	1.6%	1.02 [-0.38, 2.42]
Yamada 2019, sarcopenia, RET+PS vs CG3 Con	1.37	1.69	3	-1.09	2.27	8	1.5%	1.04 [-0.39, 2.48]
**Subtotal (95% CI)**			**227**			**342**	**100.0%**	**0.44 [0.26, 0.62]**
Heterogeneity: Chi² = 21.14, df = 12 (P = 0.05); I² = 43%								
Test for overall effect: Z = 4.87 (P < 0.00001)								
**Handgrip strength, ≥6 mo, <12 mo, fixed model**								
Dirks 2017, RET+PS vs RET+PLA-S	3.4	6.44	17	0.7	7.56	17	35.4%	0.38 [-0.30, 1.05]
Tieland 2012, RET+PS vs RET+PLA-S	2.2	4.6	31	0.45	4.6	31	64.6%	0.38 [-0.13, 0.88]
**Subtotal (95% CI)**			**48**			**48**	**100.0%**	**0.38 [-0.03, 0.78]**
Heterogeneity: Chi² = 0.00, df = 1 (P = 1.00); I² = 0%								
Test for overall effect: Z = 1.82 (P = 0.07)								
**Handgrip strength, ≥3 mo, <6 mo, fixed model**								
Dirks 2017, RET+PS vs RET+PLA-S	3.5	6	17	0.5	7.4	17	8.4%	0.43 [-0.25, 1.12]
Kim 2016, MET+PS vs CG1 MET	0.4	3.2	12	-0.2	2.4	35	9.0%	0.23 [-0.43, 0.88]
Kim 2016, MET+PS vs CG2 PS	0.4	3.2	12	0.5	3.3	34	9.0%	-0.03 [-0.69, 0.63]
Kim 2016, MET+PS vs CG3 Con	0.4	3.2	12	-0.3	2.7	34	9.0%	0.24 [-0.42, 0.90]
Rondanelli 2016, MET+PS vs PLA-S	3.2	4	69	-0.47	2.3	61	28.5%	1.10 [0.73, 1.47]
Tieland 2012, RET+PS vs RET+PLA-S	1.35	4.6	31	0	4.6	31	15.6%	0.29 [-0.21, 0.79]
Yamada 2019, dynapenia, RET+PS vs CG1 RET	0.46	1.82	6	0.21	2.26	20	4.7%	0.11 [-0.80, 1.02]
Yamada 2019, dynapenia, RET+PS vs CG2 PS	0.46	1.82	6	0.04	3.63	19	4.6%	0.12 [-0.80, 1.04]
Yamada 2019, dynapenia, RET+PS vs CG3 Con	0.46	1.82	7	-0.14	1.94	20	5.2%	0.30 [-0.56, 1.17]
Yamada 2019, sarcopenia, RET+PS vs CG1 RET	1.37	1.69	3	-0.53	2.06	8	2.0%	0.88 [-0.53, 2.28]
Yamada 2019, sarcopenia, RET+PS vs CG2 PS	1.37	1.69	3	-0.46	1.65	9	2.0%	1.02 [-0.38, 2.42]
Yamada 2019, sarcopenia, RET+PS vs CG3 Con	1.37	1.69	3	-1.09	2.27	8	1.9%	1.04 [-0.39, 2.48]
**Subtotal (95% CI)**			**181**			**296**	**100.0%**	**0.52 [0.32, 0.72]**
Heterogeneity: Chi² = 17.44, df = 11 (P = 0.10); I² = 37%								
Test for overall effect: Z = 5.15 (P < 0.00001)								
**Handgrip strength, <3 mo**								
Bjorkman 2011, RET+PS vs RET+PLA-S	3.7	27.3	46	2.2	24.5	46	100.0%	0.06 [-0.35, 0.47]
**Subtotal (95% CI)**			**46**			**46**	**100.0%**	**0.06 [-0.35, 0.47]**
Heterogeneity: Not applicable								
Test for overall effect: Z = 0.27 (P = 0.78)								

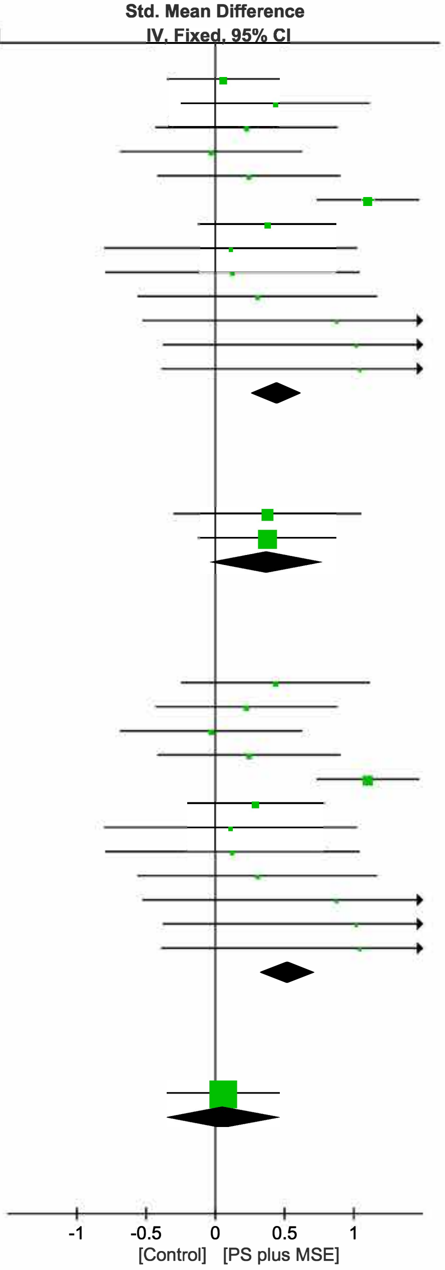

Figure S3. Forest plot summarizing effects of protein supplement (PS) plus muscle strength exercise training (MSE) on handgrip strength at an overall duration and each follow-up time point. The horizontal line links the lower and upper limits of the 95% CI of this effect. The combined effects are plotted using black diamonds. 95% CI = 95% confidence interval; Std. = standard; IV = inverse variance; CG = control group; Con = control; MET = multicomponent exercise training; PLA-S, placebo supplement; RET = resistance exercise training.

Figure S4

Study or Subgroup	PS plus MSE Mean	PS plus MSE SD	PS plus MSE Total	Control Mean	Control SD	Control Total	Weight	Std. Mean Difference IV, Random, 95% CI
**Lower extremity strength, overall, random model**								
Bonnefoy 2003, MET+PS vs PLA-S	27.8	69.4	20	-11	29.7	20	5.2%	0.71 [0.07, 1.35]
Dirks 2017, RET+PS vs RET+PLA-S	51	27.5	17	49	4.7	17	5.0%	0.10 [-0.57, 0.77]
Englund 2017, MET+PS vs MET+PLA-S	-0.04	0.19	60	0	0.19	59	6.7%	-0.21 [-0.57, 0.15]
Fiatarone 1994, RET+PS vs CG1 RET+PLA-S	14.9	9	9	8.3	8.7	25	4.5%	0.73 [-0.05, 1.52]
Fiatarone 1994, RET+PS vs CG2 PS	14.9	9	8	0.6	11.7	24	4.1%	1.25 [0.39, 2.12]
Fiatarone 1994, RET+PS vs CG3 PLA-S	14.9	9	8	1.8	10.4	26	4.1%	1.27 [0.41, 2.12]
Kim 2012, MET+PS vs CG1 MET	0.08	0.11	12	0.02	0.12	36	5.1%	0.50 [-0.16, 1.16]
Kim 2012, MET+PS vs CG2 PS	0.08	0.11	11	-0.01	0.1	37	4.9%	0.87 [0.17, 1.56]
Kim 2012, MET+PS vs CG3 Con	0.08	0.11	11	-0.14	0.1	37	4.4%	2.12 [1.31, 2.92]
Kim 2016, MET+PS vs CG1 MET	2.86	2.57	12	2.18	2.85	35	5.1%	0.24 [-0.42, 0.90]
Kim 2016, MET+PS vs CG2 PS	2.86	2.57	12	1.61	2.21	34	5.0%	0.53 [-0.13, 1.20]
Kim 2016, MET+PS vs CG3 Con	2.86	2.57	12	0.68	2.67	34	5.0%	0.81 [0.13, 1.49]
Maltais 2016, RET+EAA-PS vs RET+PLA-S	8.2	5.07	8	7.5	4.66	5	3.1%	0.13 [-0.99, 1.25]
Maltais 2016, RET+Milk-PS vs RET+PLA-S	8.6	4.16	8	7.5	4.66	5	3.1%	0.24 [-0.89, 1.36]
Molnar 2016, MET+PS vs MET	2.1	4.2	17	-1.6	3.5	17	4.8%	0.93 [0.22, 1.65]
Tieland 2012, RET+PS vs RET+PLA-S	32	16.36	31	31	16.14	31	5.9%	0.06 [-0.44, 0.56]
Yamada 2019, dynapenia, RET+PS vs CG1 RET	6.64	12.33	6	0.45	8.05	20	3.8%	0.66 [-0.27, 1.59]
Yamada 2019, dynapenia, RET+PS vs CG2 PS	6.76	11.04	6	5.91	16.64	19	3.9%	0.05 [-0.87, 0.97]
Yamada 2019, dynapenia, RET+PS vs CG3 Con	6.76	11.04	7	-8.42	16.46	20	3.9%	0.96 [0.06, 1.86]
Yamada 2019, sarcopenia, RET+PS vs CG1 RET	6.89	8.21	3	1.93	9.34	8	2.4%	0.50 [-0.85, 1.85]
Yamada 2019, sarcopenia, RET+PS vs CG2 PS	6.89	8.21	3	-1.04	12.04	9	2.5%	0.64 [-0.70, 1.99]
Yamada 2019, sarcopenia, RET+PS vs CG3 Con	6.89	8.21	3	1.67	9.92	8	2.4%	0.50 [-0.85, 1.85]
Zdzieblik 2015, RET+PS vs RET+PLA-S	17	6.58	26	7	6.86	27	5.3%	1.47 [0.85, 2.08]
**Subtotal (95% CI)**			**310**			**553**	**100.0%**	**0.65 [0.39, 0.90]**
Heterogeneity: Tau² = 0.22; Chi² = 57.43, df = 22 (P < 0.0001); I² = 62%								
Test for overall effect: Z = 4.95 (P < 0.00001)								
**Lower extremity strength, ≥6 mo, <12 mo, fixed model**								
Bonnefoy 2003, MET+PS vs PLA-S	52.4	97.8	20	16.83	79.5	20	15.5%	0.39 [-0.24, 1.02]
Dirks 2017, RET+PS vs RET+PLA-S	51	27.5	17	49	4.7	17	13.4%	0.10 [-0.57, 0.77]
Englund 2017, MET+PS vs MET+PLA-S	-0.04	0.19	60	0	0.19	59	46.7%	-0.21 [-0.57, 0.15]
Tieland 2012, RET+PS vs RET+PLA-S	45	16.4	31	46	16.6	31	24.5%	-0.06 [-0.56, 0.44]
**Subtotal (95% CI)**			**128**			**127**	**100.0%**	**-0.04 [-0.28, 0.21]**
Heterogeneity: Chi² = 2.84, df = 3 (P = 0.42); I² = 0%								
Test for overall effect: Z = 0.31 (P = 0.76)								
**Lower extremity strength, ≥3 mo, <6 mo, random model**								
Bonnefoy 2003, MET+PS vs PLA-S	27.8	69.4	20	-11	29.7	20	6.4%	0.71 [0.07, 1.35]
Dirks 2017, RET+PS vs RET+PLA-S	31	17.1	17	39	4.4	17	6.1%	-0.63 [-1.32, 0.06]
Kim 2012, MET+PS vs CG1 MET	0.08	0.11	12	0.02	0.12	36	6.3%	0.50 [-0.16, 1.16]
Kim 2012, MET+PS vs CG2 PS	0.08	0.11	11	-0.01	0.1	37	6.1%	0.87 [0.17, 1.56]
Kim 2012, MET+PS vs CG3 Con	0.08	0.11	11	-0.14	0.1	37	5.4%	2.12 [1.31, 2.92]
Kim 2016, MET+PS vs CG1 MET	2.86	2.57	12	2.18	2.85	35	6.3%	0.24 [-0.42, 0.90]
Kim 2016, MET+PS vs CG2 PS	2.86	2.57	12	1.61	2.21	34	6.2%	0.53 [-0.13, 1.20]
Kim 2016, MET+PS vs CG3 Con	2.86	2.57	12	0.68	2.67	34	6.2%	0.81 [0.13, 1.49]
Maltais 2016, RET+EAA-PS vs RET+PLA-S	8.2	5.07	8	7.5	4.66	5	3.9%	0.13 [-0.99, 1.25]
Maltais 2016, RET+Milk-PS vs RET+PLA-S	8.6	4.16	8	7.5	4.66	5	3.8%	0.24 [-0.89, 1.36]
Molnar 2016, MET+PS vs MET	2.1	4.2	17	-1.6	3.5	17	6.0%	0.93 [0.22, 1.65]
Tieland 2012, RET+PS vs RET+PLA-S	32	16.36	31	31	16.14	31	7.4%	0.06 [-0.44, 0.56]
Yamada 2019, dynapenia, RET+PS vs CG1 RET	6.64	12.33	6	0.45	8.05	20	4.7%	0.66 [-0.27, 1.59]
Yamada 2019, dynapenia, RET+PS vs CG2 PS	6.76	11.04	6	5.91	16.64	19	4.8%	0.05 [-0.87, 0.97]
Yamada 2019, dynapenia, RET+PS vs CG3 Con	6.76	11.04	7	-8.42	16.46	20	4.9%	0.96 [0.06, 1.86]
Yamada 2019, sarcopenia, RET+PS vs CG1 RET	6.89	8.21	3	1.93	9.34	8	3.0%	0.50 [-0.85, 1.85]
Yamada 2019, sarcopenia, RET+PS vs CG2 PS	6.89	8.21	3	-1.04	12.04	9	3.1%	0.64 [-0.70, 1.99]
Yamada 2019, sarcopenia, RET+PS vs CG3 Con	6.89	8.21	3	1.67	9.92	8	3.0%	0.50 [-0.85, 1.85]
Zdzieblik 2015, RET+PS vs RET+PLA-S	17	6.58	26	7	6.86	27	6.6%	1.47 [0.85, 2.08]
**Subtotal (95% CI)**			**225**			**419**	**100.0%**	**0.60 [0.32, 0.89]**
Heterogeneity: Tau² = 0.22; Chi² = 44.18, df = 18 (P = 0.0005); I² = 59%								
Test for overall effect: Z = 4.15 (P < 0.0001)								
**Lower extremity strength, <3 mo, fixed model**								
Fiatarone 1994, RET+PS vs CG1 RET+PLA-S	14.9	9	9	8.3	8.7	25	37.5%	0.73 [-0.05, 1.52]
Fiatarone 1994, RET+PS vs CG2 PS	14.9	9	8	0.6	11.7	24	30.9%	1.25 [0.39, 2.12]
Fiatarone 1994, RET+PS vs CG3 PLA-S	14.9	9	8	1.8	10.4	26	31.6%	1.27 [0.41, 2.12]
**Subtotal (95% CI)**			**25**			**75**	**100.0%**	**1.06 [0.58, 1.54]**
Heterogeneity: Chi² = 1.07, df = 2 (P = 0.58); I² = 0%								
Test for overall effect: Z = 4.33 (P < 0.0001)								

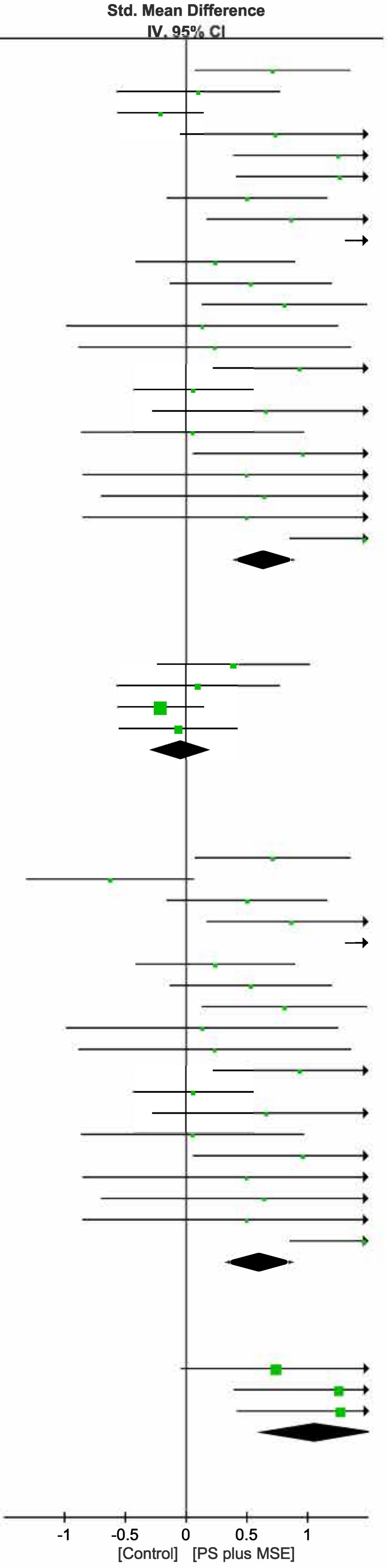

Figure S4. Forest plot summarizing effects of protein supplement (PS) plusmuscle strength exercise training (MSE) on leg strength at an overall duration and each follow-up time point. The horizontal line links the lower and upper limits of the 95% CI of this effect. The combined effects are plotted using black diamonds. 95% CI = 95% confidence interval; Std. = standard; IV = inverse variance; CG = control group; Con = control; MET = multicomponent exercise training; PLA-S, placebo supplement; RET = resistance exercise training.

Figure S5

Study or Subgroup	PS plus MSE Mean	PS plus MSE SD	PS plus MSE Total	Control Mean	Control SD	Control Total	Weight	Std. Mean Difference IV, Fixed, 95% CI
**Walk ability, overall FU duration, random model**								
Bjorkman 2011, RET+PS vs RET+PLA-S	0.4	12.7	46	2.7	12.53	46	7.9%	-0.18 [-0.59, 0.23]
Bonnefoy 2003, MET+PS vs PLA-S	20.5	6.29	22	15.8	7.17	22	5.4%	0.68 [0.07, 1.29]
Bonnefoy 2012, MET+PS vs Con	0.01	0.19	53	-0.02	0.24	49	8.2%	0.14 [-0.25, 0.53]
Dirks 2017, RET+PS vs RET+PLA-S	0	1.8	17	0.1	1.01	17	4.8%	-0.07 [-0.74, 0.61]
Fiatarone 1994, RET+PS vs CG1 RET+PLA-S	14.9	26.7	9	8.6	25.8	25	4.1%	0.24 [-0.53, 1.00]
Fiatarone 1994, RET+PS vs CG2 PS	14.9	26.7	8	5.2	26.9	24	3.8%	0.35 [-0.45, 1.16]
Fiatarone 1994, RET+PS vs CG3 PLA-S	14.9	26.7	8	-7.2	25.9	26	3.7%	0.83 [0.01, 1.65]
Fielding 2017, MET+PS vs MET+PLA-S	0.1	0.11	67	0.1	0.1	68	9.0%	0.00 [-0.34, 0.34]
Kim 2012, MET+PS vs CG1 MET	0.28	0.23	12	0.32	0.17	36	5.0%	-0.21 [-0.87, 0.44]
Kim 2012, MET+PS vs CG2 PS	0.28	0.23	11	0.21	0.17	37	4.8%	0.37 [-0.30, 1.05]
Kim 2012, MET+PS vs CG3 Con	0.28	0.23	11	0.07	0.2	37	4.6%	1.00 [0.29, 1.70]
Kim 2016, MET+PS vs CG1 MET	5.7	12.41	12	1.05	12.37	35	4.9%	0.37 [-0.29, 1.03]
Kim 2016, MET+PS vs CG2 PS	5.7	12.41	12	2.63	8.53	34	4.9%	0.31 [-0.35, 0.97]
Kim 2016, MET+PS vs CG3 Con	5.7	12.41	12	2.35	9.6	34	4.9%	0.32 [-0.34, 0.98]
Maltais 2016, RET+EAA-PS vs RET+PLA-S	0.3	0.19	8	-0.1	0.32	5	1.8%	1.52 [0.20, 2.83]
Maltais 2016, RET+Milk-PS vs RET+PLA-S	0.1	0.13	8	-0.1	0.32	5	2.1%	0.85 [-0.33, 2.03]
Tieland 2012, RET+PS vs RET+PLA-S	0	1.21	31	-0.2	1.12	31	6.7%	0.17 [-0.33, 0.67]
Yamada 2019, dynapenia, RET+PS vs CG1 RET	0.62	1.31	6	0.09	1.87	20	3.2%	0.29 [-0.63, 1.21]
Yamada 2019, dynapenia, RET+PS vs CG2 PS	0.62	1.31	6	0.75	2.12	19	3.2%	-0.06 [-0.98, 0.85]
Yamada 2019, dynapenia, RET+PS vs CG3 Con	0.62	1.31	7	-0.03	1.06	20	3.4%	0.56 [-0.32, 1.44]
Yamada 2019, sarcopenia, RET+PS vs CG1 RET	1.88	0.44	3	0.73	1.46	8	1.6%	0.81 [-0.59, 2.20]
Yamada 2019, sarcopenia, RET+PS vs CG2 PS	1.88	0.44	3	0.18	0.75	9	1.1%	2.24 [0.54, 3.95]
Yamada 2019, sarcopenia, RET+PS vs CG3 Con	1.88	0.44	3	-0.43	0.79	8	0.8%	2.91 [0.89, 4.92]
**Subtotal (95% CI)**			**375**			**615**	**100.0%**	**0.33 [0.14, 0.52]**
Heterogeneity: Tau² = 0.07; Chi² = 36.13, df = 22 (P = 0.03); I² = 39%								
Test for overall effect: Z = 3.44 (P = 0.0006)								
**Walk ability, ≥6 mo, <12 mo, fixed model**								
Bonnefoy 2003, MET+PS vs PLA-S	20.5	6.29	22	15.8	7.17	22	15.3%	0.68 [0.07, 1.29]
Dirks 2017, RET+PS vs RET+PLA-S	0.2	1.69	17	0.4	0.96	17	12.6%	-0.14 [-0.82, 0.53]
Fielding 2017, MET+PS vs MET+PLA-S	0.08	0.12	68	0.11	0.12	66	49.2%	-0.25 [-0.59, 0.09]
Tieland 2012, RET+PS vs RET+PLA-S	0.1	1.91	31	0.2	1.77	31	22.9%	-0.05 [-0.55, 0.44]
**Subtotal (95% CI)**			**138**			**136**	**100.0%**	**-0.05 [-0.29, 0.19]**
Heterogeneity: Chi² = 6.96, df = 3 (P = 0.07); I² = 57%								
Test for overall effect: Z = 0.39 (P = 0.70)								
**Walk ability, ≥3 mo, <6 mo, fixed model**								
Bonnefoy 2003, MET+PS vs PLA-S	10.14	8.64	22	6.5	8.35	22	6.4%	0.42 [-0.18, 1.02]
Bonnefoy 2012, MET+PS vs Con	0.01	0.19	53	-0.02	0.24	49	15.1%	0.14 [-0.25, 0.53]
Dirks 2017, RET+PS vs RET+PLA-S	0	1.8	17	0.1	1.01	17	5.0%	-0.07 [-0.74, 0.61]
Fielding 2017, MET+PS vs MET+PLA-S	0.1	0.11	67	0.1	0.1	68	20.0%	0.00 [-0.34, 0.34]
Kim 2012, MET+PS vs CG1 MET	0.28	0.23	12	0.32	0.17	36	5.3%	-0.21 [-0.87, 0.44]
Kim 2012, MET+PS vs CG2 PS	0.28	0.23	11	0.21	0.17	37	5.0%	0.37 [-0.30, 1.05]
Kim 2012, MET+PS vs CG3 Con	0.28	0.23	11	0.07	0.2	37	4.6%	1.00 [0.29, 1.70]
Kim 2016, MET+PS vs CG1 MET	5.7	12.41	12	1.05	12.37	35	5.2%	0.37 [-0.29, 1.03]
Kim 2016, MET+PS vs CG2 PS	5.7	12.41	12	2.63	8.53	34	5.2%	0.31 [-0.35, 0.97]
Kim 2016, MET+PS vs CG3 Con	5.7	12.41	12	2.35	9.6	34	5.2%	0.32 [-0.34, 0.98]
Maltais 2016, RET+EAA-PS vs RET+PLA-S	0.3	0.19	8	-0.1	0.32	5	1.3%	1.52 [0.20, 2.83]
Maltais 2016, RET+Milk-PS vs RET+PLA-S	0.1	0.13	8	-0.1	0.32	5	1.6%	0.85 [-0.33, 2.03]
Tieland 2012, RET+PS vs RET+PLA-S	0	1.21	31	-0.2	1.12	31	9.2%	0.17 [-0.33, 0.67]
Yamada 2019, dynapenia, RET+PS vs CG1 RET	0.62	1.31	6	0.09	1.87	20	2.7%	0.29 [-0.63, 1.21]
Yamada 2019, dynapenia, RET+PS vs CG2 PS	0.62	1.31	6	0.75	2.12	19	2.7%	-0.06 [-0.98, 0.85]
Yamada 2019, dynapenia, RET+PS vs CG3 Con	0.62	1.31	7	-0.03	1.06	20	3.0%	0.56 [-0.32, 1.44]
Yamada 2019, sarcopenia, RET+PS vs CG1 RET	1.88	0.44	3	0.73	1.46	8	1.2%	0.81 [-0.59, 2.20]
Yamada 2019, sarcopenia, RET+PS vs CG2 PS	1.88	0.44	3	0.18	0.75	9	0.8%	2.24 [0.54, 3.95]
Yamada 2019, sarcopenia, RET+PS vs CG3 Con	1.88	0.44	3	-0.43	0.79	8	0.6%	2.91 [0.89, 4.92]
**Subtotal (95% CI)**			**304**			**494**	**100.0%**	**0.26 [0.11, 0.42]**
Heterogeneity: Chi² = 28.24, df = 18 (P = 0.06); I² = 36%								
Test for overall effect: Z = 3.44 (P = 0.0006)								
**Walk ability, <3 mo, fixed model**								
Bjorkman 2011, RET+PS vs RET+PLA-S	0.4	12.7	46	2.7	12.53	46	55.7%	-0.18 [-0.59, 0.23]
Fiatarone 1994, RET+PS vs CG1 RET+PLA-S	14.9	26.7	9	8.6	25.8	25	16.0%	0.24 [-0.53, 1.00]
Fiatarone 1994, RET+PS vs CG2 PS	14.9	26.7	8	5.2	26.9	24	14.4%	0.35 [-0.45, 1.16]
Fiatarone 1994, RET+PS vs CG3 PLA-S	14.9	26.7	8	-7.2	25.9	26	13.9%	0.83 [0.01, 1.65]
**Subtotal (95% CI)**			**71**			**121**	**100.0%**	**0.10 [-0.20, 0.41]**
Heterogeneity: Chi² = 5.33, df = 3 (P = 0.15); I² = 44%								
Test for overall effect: Z = 0.66 (P = 0.51)								

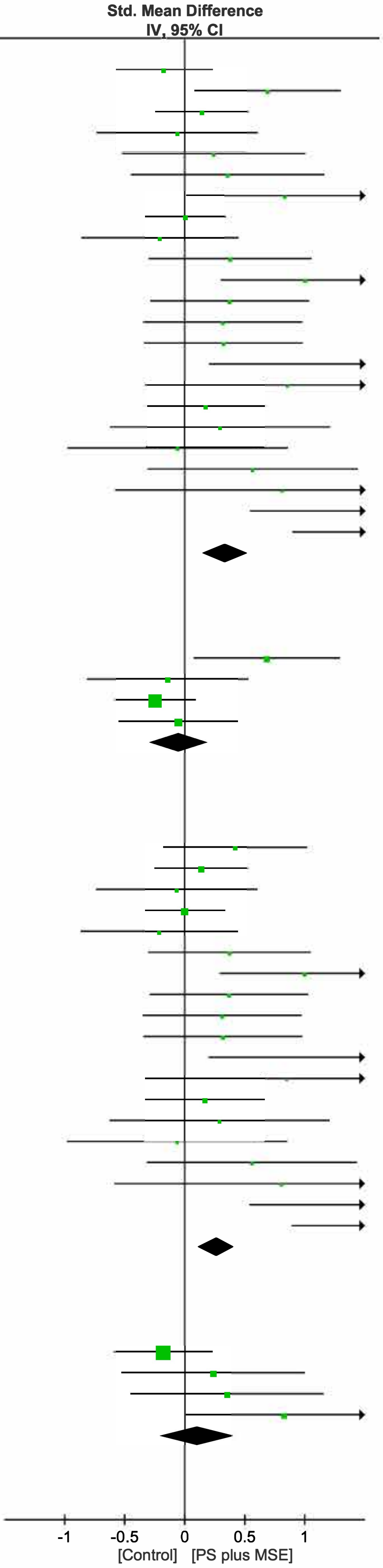

Figure S5. Forest plot summarizing effects of protein supplement (PS) plusmuscle strength exercise training (MSE) onwalk capability at an overall duration andeach follow-up time point. The horizontal line links the lower and upper limits of the 95% CI of this effect. The combined effects are plotted using black diamonds. 95% CI = 95% confidence interval; Std. = standard; IV = inverse variance; CG = control group; Con = control; MET = multicomponent exercise training; PLA-S, placebo supplement; RET = resistance exercise training.

Figure S6

Study or Subgroup	PS plus MSE Mean	PS plus MSE SD	PS plus MSE Total	Control Mean	Control SD	Control Total	Weight	Std. Mean Difference IV, Fixed, 95% CI
**hair rise, Overall, fixed model**								
Bjorkman 2011, RET+PS vs RET+PLA-S	4	22.68	46	7.8	14.73	46	23.1%	-0.20 [-0.61, 0.21]
Bonnefoy 2003, MET+PS vs PLA-S	0.68	0.59	22	0.22	1.05	22	10.7%	0.53 [-0.07, 1.13]
Bonnefoy 2012, MET+PS vs Con	7.7	23.19	23	0	23.78	49	15.6%	0.32 [-0.18, 0.82]
Dirks 2017, RET+PS vs RET+PLA-S	2.8	2.5	17	3.9	3.46	17	8.4%	-0.36 [-1.03, 0.32]
Maltais 2016, RET+EAA-PS vs RET+PLA-S	1.2	1.64	8	0.4	1.36	5	3.0%	0.48 [-0.66, 1.62]
Maltais 2016, RET+Milk-PS vs RET+PLA-S	0.1	1.76	8	0.4	1.36	5	3.1%	-0.17 [-1.29, 0.95]
Tieland 2012, RET+PS vs RET+PLA-S	1.95	4.54	31	0.9	4.4	31	15.6%	0.23 [-0.27, 0.73]
Yamada 2019, dynapenia, RET+PS vs CG1 RET	1.71	4.31	6	0.95	4.63	20	4.7%	0.16 [-0.75, 1.07]
Yamada 2019, dynapenia, RET+PS vs CG2 PS	1.71	4.31	6	0.68	6.08	19	4.6%	0.17 [-0.75, 1.09]
Yamada 2019, dynapenia, RET+PS vs CG3 Con	1.71	4.31	7	-1	1.92	20	4.7%	0.98 [0.07, 1.88]
Yamada 2019, sarcopenia, RET+PS vs CG1 RET	1.31	1.29	3	0.79	1.15	8	2.2%	0.40 [-0.94, 1.75]
Yamada 2019, sarcopenia, RET+PS vs CG2 PS	1.31	1.29	3	0.67	0.82	9	2.2%	0.63 [-0.71, 1.98]
Yamada 2019, sarcopenia, RET+PS vs CG3 Con	1.31	1.29	3	0.43	1.41	8	2.1%	0.58 [-0.78, 1.94]
**Subtotal (95% CI)**			**183**			**259**	**100.0%**	**0.17 [-0.02, 0.37]**
Heterogeneity: Chi² = 11.79, df = 12 (P = 0.46); I² = 0%								
Test for overall effect: Z = 1.72 (P = 0.09)								
**Chair rise, ≥6 mo, <12 mo, random model**								
Bonnefoy 2003, MET+PS vs PLA-S	0.68	0.59	22	0.22	1.05	22	33.0%	0.53 [-0.07, 1.13]
Dirks 2017, RET+PS vs RET+PLA-S	2.8	2.5	17	3.9	3.46	17	30.5%	-0.36 [-1.03, 0.32]
Tieland 2012, RET+PS vs RET+PLA-S	2.1	4.6	31	4.15	4.54	31	36.5%	-0.44 [-0.95, 0.06]
**Subtotal (95% CI)**			**70**			**70**	**100.0%**	**-0.09 [-0.71, 0.52]**
Heterogeneity: Tau² = 0.21; Chi² = 6.52, df = 2 (P = 0.04); I² = 69%								
Test for overall effect: Z = 0.30 (P = 0.76)								
**Chair rise, ≥3 mo, <6 mo, fixed model**								
Bonnefoy 2012, MET+PS vs Con	7.7	23.19	23	0	23.78	49	23.7%	0.32 [-0.18, 0.82]
Dirks 2017, RET+PS vs RET+PLA-S	0.2	6.44	17	2.5	2.95	17	12.7%	-0.45 [-1.13, 0.23]
Maltais 2016, RET+EAA-PS vs RET+PLA-S	1.2	1.64	8	0.4	1.36	5	4.5%	0.48 [-0.66, 1.62]
Maltais 2016, RET+Milk-PS vs RET+PLA-S	0.1	1.76	8	0.4	1.36	5	4.7%	-0.17 [-1.29, 0.95]
Tieland 2012, RET+PS vs RET+PLA-S	1.95	4.54	31	0.9	4.4	31	23.6%	0.23 [-0.27, 0.73]
Yamada 2019, dynapenia, RET+PS vs CG1 RET	1.71	4.31	6	0.95	4.63	20	7.0%	0.16 [-0.75, 1.07]
Yamada 2019, dynapenia, RET+PS vs CG2 PS	1.71	4.31	6	0.68	6.08	19	7.0%	0.17 [-0.75, 1.09]
Yamada 2019, dynapenia, RET+PS vs CG3 Con	1.71	4.31	7	-1	1.92	20	7.2%	0.98 [0.07, 1.88]
Yamada 2019, sarcopenia, RET+PS vs CG1 RET	1.31	1.29	3	0.79	1.15	8	3.3%	0.40 [-0.94, 1.75]
Yamada 2019, sarcopenia, RET+PS vs CG2 PS	1.31	1.29	3	0.67	0.82	9	3.3%	0.63 [-0.71, 1.98]
Yamada 2019, sarcopenia, RET+PS vs CG3 Con	1.31	1.29	3	0.43	1.41	8	3.2%	0.58 [-0.78, 1.94]
**Subtotal (95% CI)**			**115**			**191**	**100.0%**	**0.23 [-0.01, 0.48]**
Heterogeneity: Chi² = 7.92, df = 10 (P = 0.64); I² = 0%								
Test for overall effect: Z = 1.89 (P = 0.06)								
**Chair rise, <3 mo**								
Bjorkman 2011, RET+PS vs RET+PLA-S	4	22.68	46	7.8	14.73	46	100.0%	-0.20 [-0.61, 0.21]
**Subtotal (95% CI)**			**46**			**46**	**100.0%**	**-0.20 [-0.61, 0.21]**
Heterogeneity: Not applicable								
Test for overall effect: Z = 0.94 (P = 0.35)								

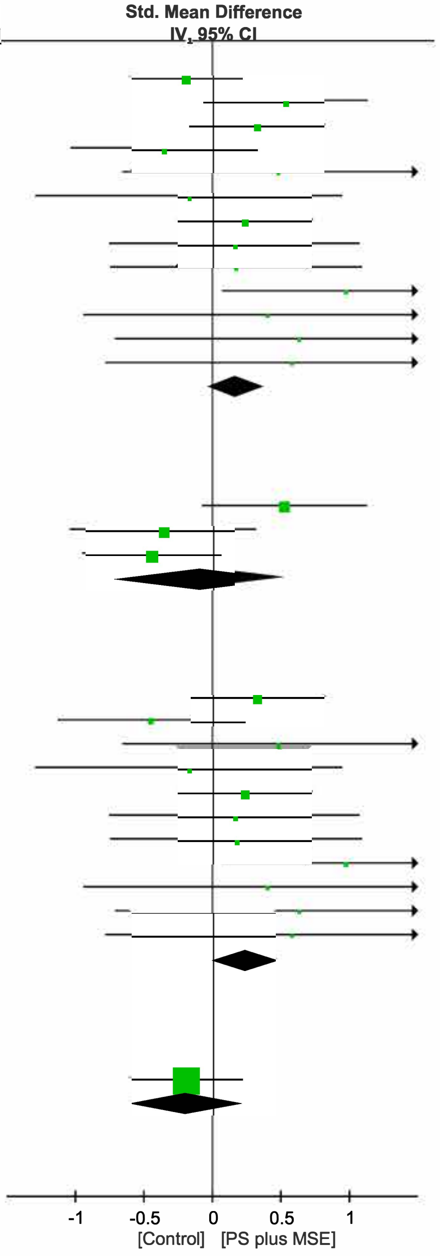

Figure S6. Forest plot summarizing effects of protein supplement (PS) plus muscle strength exercise training (MSE) on chair rise at an overall duration and each follow-up time point. The horizontal line links the lower and upper limits of the 95% CI of this effect. The combined effects are plotted using black diamonds. 95% CI = 95% confidence interval; Std. = standard; Iv = inverse variance; CG = control group; Con = control; MET = multicomponent exercise training; PLA-S, placebo supplement; RET = resistance exercise training.

Figure S7

Study or Subgroup	PS plus MSE Mean	PS plus MSE SD	PS plus MSE Total	Control Mean	Control SD	Control Total	Weight	Std. Mean Difference IV, Fixed, 95% CI
**Stair climb, Overall**								
Bonnefoy 2003, MET+PS vs PLA-S	0.02	0.45	22	-0.1	0.49	22	19.9%	0.25 [-0.34, 0.84]
Bonnefoy 2012, MET+PS vs Con	0	12.37	53	0	30.89	49	46.6%	0.00 [-0.39, 0.39]
Fiatarone 1994, RET+PS vs CG1 RET+PLA-S	7.9	13.5	9	11.1	12.5	25	12.0%	-0.24 [-1.01, 0.52]
Fiatarone 1994, RET+PS vs CG2 PS	7.9	13.5	8	4.2	13.2	24	10.9%	0.27 [-0.53, 1.08]
Fiatarone 1994, RET+PS vs CG3 PLA-S	7.9	13.5	8	-2.5	13.7	26	10.6%	0.74 [-0.07, 1.56]
**Subtotal (95% CI)**			**100**			**146**	**100.0%**	**0.13 [-0.14, 0.39]**
Heterogeneity: Chi² = 3.81, df = 4 (P = 0.43); I² = 0%								
Test for overall effect: Z = 0.95 (P = 0.34)								
**Stair climb, ≥6 mo, <12 mo**								
Bonnefoy 2003, MET+PS vs PLA-S	0.14	0.33	22	0.1	0.36	22	100.0%	0.11 [-0.48, 0.71]
**Subtotal (95% CI)**			**22**			**22**	**100.0%**	**0.11 [-0.48, 0.71]**
Heterogeneity: Not applicable								
Test for overall effect: Z = 0.38 (P = 0.71)								
**Stair climb, ≥3 mo, <6 mo**								
Bonnefoy 2003, MET+PS vs PLA-S	0.02	0.45	22	-0.1	0.49	22	30.0%	0.25 [-0.34, 0.84]
Bonnefoy 2012, MET+PS vs Con	0	12.37	53	0	30.89	49	70.0%	0.00 [-0.39, 0.39]
**Subtotal (95% CI)**			**75**			**71**	**100.0%**	**0.08 [-0.25, 0.40]**
Heterogeneity: Chi² = 0.48, df = 1 (P = 0.49); I² = 0%								
Test for overall effect: Z = 0.45 (P = 0.65)								
**Stair climb, <3 mo**								
Fiatarone 1994, RET+PS vs CG1 RET+PLA-S	7.9	13.5	9	11.1	12.5	25	35.9%	-0.24 [-1.01, 0.52]
Fiatarone 1994, RET+PS vs CG2 PS	7.9	13.5	8	4.2	13.2	24	32.5%	0.27 [-0.53, 1.08]
Fiatarone 1994, RET+PS vs CG3 PLA-S	7.9	13.5	8	-2.5	13.7	26	31.6%	0.74 [-0.07, 1.56]
**Subtotal (95% CI)**			**25**			**75**	**100.0%**	**0.24 [-0.22, 0.69]**
Heterogeneity: Chi² = 3.02, df = 2 (P = 0.22); I² = 34%								
Test for overall effect: Z = 1.01 (P = 0.31)								

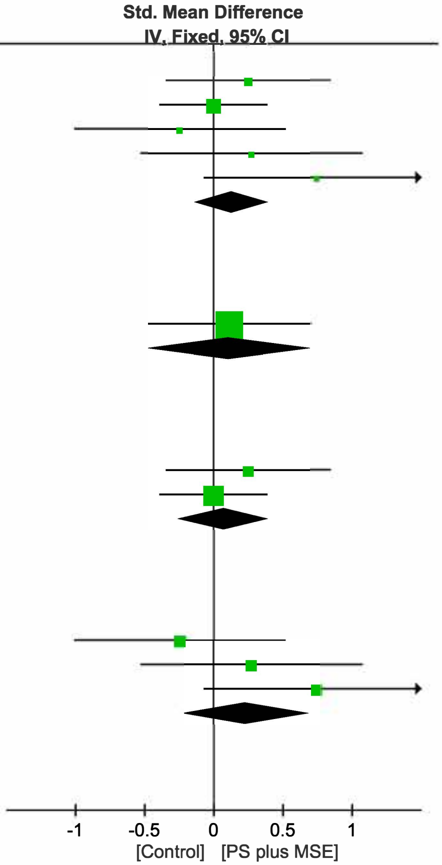

Figure S7. Forest plot summarizing effects of protein supplement (PS) plus muscle strength exercise training (MSE) on stair climb at an overall duration and each follow-up time point. The horizontal line links the lower and upper limits of the 95% CI of this effect. The combined effects are plotted using black diamonds. 95% CI = 95% confidence interval; Std. = standard; IV = inverse variance; CG = control group; Con = control; MET = multicomponent exercise training; PLA-S, placebo supplement; RET = resistance exercise training.

Figure S8

Study or Subgroup	PS plus MSE Mean	PS plus MSE SD	PS plus MSE Total	Control Mean	Control SD	Control Total	Weight	Std. Mean Difference IV, Fixed, 95% CI
**TUG, Overall**								
Bonnefoy 2012, MET+PS vs Con	10	128	23	0	20	49	75.1%	0.13 [-0.36, 0.63]
Maltais 2016, RET+EAA-PS vs RET+PLA-S	1.3	0.79	8	-0.1	0.84	5	10.3%	1.61 [0.27, 2.95]
Maltais 2016, RET+Milk-PS vs RET+PLA-S	0.1	0.72	8	-0.1	0.84	5	14.6%	0.24 [-0.88, 1.37]
**Subtotal (95% CI)**			**39**			**59**	**100.0%**	**0.30 [-0.13, 0.73]**
Heterogeneity: Chi^2 = 4.11, df = 2 (P = 0.13); I^2 = 51%								
Test for overall effect: Z = 1.38 (P = 0.17)								
**TUG, ≥6 mo, <12 mo**								
**Subtotal (95% CI)**			**0**			**0**		**Not estimable**
Heterogeneity: Not applicable								
Test for overall effect: Not applicable								
**TUG, ≥3 mo, <6 mo**								
Bonnefoy 2012, MET+PS vs Con	10	128	23	0	20	49	75.1%	0.13 [-0.36, 0.63]
Maltais 2016, RET+EAA-PS vs RET+PLA-S	1.3	0.79	8	-0.1	0.84	5	10.3%	1.61 [0.27, 2.95]
Maltais 2016, RET+Milk-PS vs RET+PLA-S	0.1	0.72	8	-0.1	0.84	5	14.6%	0.24 [-0.88, 1.37]
**Subtotal (95% CI)**			**39**			**59**	**100.0%**	**0.30 [-0.13, 0.73]**
Heterogeneity: Chi^2 = 4.11, df = 2 (P = 0.13); I^2 = 51%								
Test for overall effect: Z = 1.38 (P = 0.17)								
**TUG, <3 mo**								
**Subtotal (95% CI)**			**0**			**0**		**Not estimable**
Heterogeneity: Not applicable								
Test for overall effect: Not applicable								

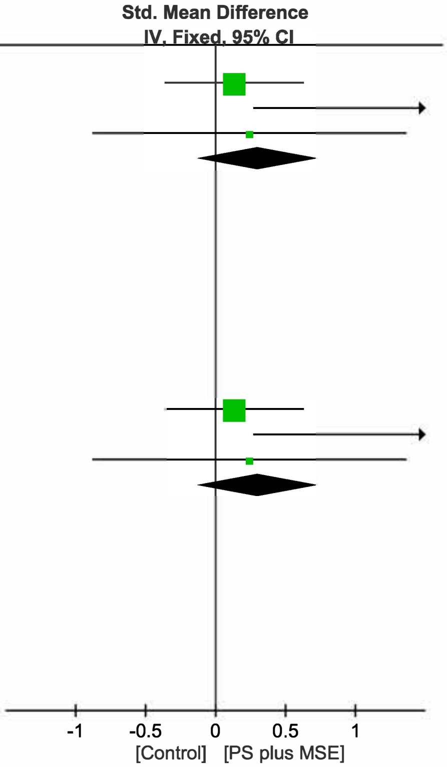

Figure S8. Forest plot summarizing effects of protein supplement (PS) plus muscle strength exercise training (MSE) on timed up-and-go (TUG) at an overall duration and each follow-up time point. The horizontal line links the lower and upper limits of the 95% CI of this effect. The combined effects are plotted using black diamonds. 95% CI = 95% confidence interval; Std. = standard; IV = inverse variance; CG = control group; Con = control; MET = multicomponent exercise training; PLA-S, placebo supplement; RET = resistance exercise training.

Figure S9

Study or Subgroup	PS plus MSE Mean	PS plus MSE SD	PS plus MSE Total	Control Mean	Control SD	Control Total	Weight	Std. Mean Difference IV, Fixed, 95% CI
**SPPB, overall**								
Dirks 2017, RET+PS vs RET+PLA-S	1.2	1.7	17	1	1.4	17	14.9%	0.13 [-0.55, 0.80]
Fielding 2017, MET+PS vs MET+PLA-S	1.8	2.78	67	2.3	1.97	68	58.9%	-0.21 [-0.54, 0.13]
Tieland 2012, RET+PS vs RET+PLA-S	1.2	1.51	31	0.4	1.55	31	26.3%	0.52 [0.01, 1.02]
**Subtotal (95% CI)**			**115**			**116**	**100.0%**	**0.03 [-0.23, 0.29]**
Heterogeneity: Chi² = 5.50, df = 2 (P = 0.06); I² = 64%								
Test for overall effect: Z = 0.25 (P = 0.81)								
**SPPB, ≥6 mo, <12 mo**								
Dirks 2017, RET+PS vs RET+PLA-S	1.5	1.6	17	1.7	1.5	17	14.9%	-0.13 [-0.80, 0.55]
Fielding 2017, MET+PS vs MET+PLA-S	2.1	1.36	68	2.65	1.36	66	57.8%	-0.40 [-0.74, -0.06]
Tieland 2012, RET+PS vs RET+PLA-S	1.5	1.47	31	1.3	1.55	31	27.2%	0.13 [-0.37, 0.63]
**Subtotal (95% CI)**			**116**			**114**	**100.0%**	**-0.22 [-0.48, 0.04]**
Heterogeneity: Chi² = 3.06, df = 2 (P = 0.22); I² = 35%								
Test for overall effect: Z = 1.62 (P = 0.10)								
**SPPB, ≥3 mo, <6 mo**								
Dirks 2017, RET+PS vs RET+PLA-S	1.2	1.7	17	1	1.4	17	14.9%	0.13 [-0.55, 0.80]
Fielding 2017, MET+PS vs MET+PLA-S	1.8	2.78	67	2.3	1.97	68	58.9%	-0.21 [-0.54, 0.13]
Tieland 2012, RET+PS vs RET+PLA-S (1)	1.2	1.51	31	0.4	1.55	31	26.3%	0.52 [0.01, 1.02]
**Subtotal (95% CI)**			**115**			**116**	**100.0%**	**0.03 [-0.23, 0.29]**
Heterogeneity: Chi² = 5.50, df = 2 (P = 0.06); I² = 64%								
Test for overall effect: Z = 0.25 (P = 0.81)								
**SPPB, <3 mo**								
**Subtotal (95% CI)**			**0**			**0**		**Not estimable**
Heterogeneity: Not applicable								
Test for overall effect: Not applicable								

Std. Mean Difference
IV, Fixed, 95% CI

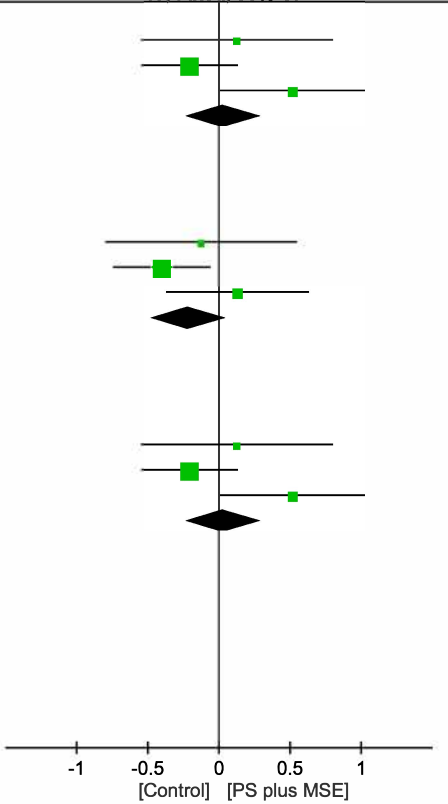

Figure S9. Forest plot summarizing effects of protein supplement (PS) plus muscle strength exercise training (MSE) on SPPB at an overall duration and each follow-up time point. The horizontal line links the lower and upper limits of the 95% CI of this effect. The combined effects are plotted using black diamonds. 95% CI = 95% confidence interval; Std. = standard; IV = inverse variance; CG = control group; Con = control; MET = multicomponent exercise training; PLA-S, placebo supplement; RET = resistance exercise training; SPPB = short physical performance battery.

Figure S10

Study or Subgroup	PS plus MSET Mean	PS plus MSET SD	PS plus MSET Total	Control Mean	Control SD	Control Total	Weight	Std. Mean Difference IV, Fixed, 95% CI
**Single-leg stance, Overall**								
Yamada 2019, dynapenia, RET+PS vs CG1 RET	0.76	4.11	6	-0.14	10.93	20	22.4%	0.09 [-0.82, 1.00]
Yamada 2019, dynapenia, RET+PS vs CG2 PS	0.76	4.11	6	0.29	7.55	19	22.2%	0.07 [-0.85, 0.98]
Yamada 2019, dynapenia, RET+PS vs CG3 Con	0.76	4.11	7	2.13	5.79	20	25.0%	-0.24 [-1.11, 0.62]
Yamada 2019, sarcopenia, RET+PS vs CG1 RET	2.43	4.03	3	0.3	2.4	8	9.9%	0.68 [-0.69, 2.06]
Yamada 2019, sarcopenia, RET+PS vs CG2 PS	2.43	4.03	3	-0.87	3.42	9	9.9%	0.86 [-0.51, 2.23]
Yamada 2019, sarcopenia, RET+PS vs CG3 Con	2.43	4.03	3	1.56	5.15	8	10.6%	0.16 [-1.17, 1.49]
**Subtotal (95% CI)**			**28**			**84**	**100.0%**	**0.14 [-0.29, 0.58]**
Heterogeneity: Chi² = 2.46, df = 5 (P = 0.78); I² = 0%								
Test for overall effect: Z = 0.65 (P = 0.52)								
**Single-leg stance, ≥6 mo, <12 mo**								
**Subtotal (95% CI)**			**0**			**0**		**Not estimable**
Heterogeneity: Not applicable								
Test for overall effect: Not applicable								
**Single-leg stance, ≥3 mo, <6 mo**								
Yamada 2019, dynapenia, RET+PS vs CG1 RET	0.76	4.11	6	-0.14	10.93	20	22.4%	0.09 [-0.82, 1.00]
Yamada 2019, dynapenia, RET+PS vs CG2 PS	0.76	4.11	6	0.29	7.55	19	22.2%	0.07 [-0.85, 0.98]
Yamada 2019, dynapenia, RET+PS vs CG3 Con	0.76	4.11	7	2.13	5.79	20	25.0%	-0.24 [-1.11, 0.62]
Yamada 2019, sarcopenia, RET+PS vs CG1 RET	2.43	4.03	3	0.3	2.4	8	9.9%	0.68 [-0.69, 2.06]
Yamada 2019, sarcopenia, RET+PS vs CG2 PS	2.43	4.03	3	-0.87	3.42	9	9.9%	0.86 [-0.51, 2.23]
Yamada 2019, sarcopenia, RET+PS vs CG3 Con	2.43	4.03	3	1.56	5.15	8	10.6%	0.16 [-1.17, 1.49]
**Subtotal (95% CI)**			**28**			**84**	**100.0%**	**0.14 [-0.29, 0.58]**
Heterogeneity: Chi² = 2.46, df = 5 (P = 0.78); I² = 0%								
Test for overall effect: Z = 0.65 (P = 0.52)								
**Single-leg stance, <3 mo**								
**Subtotal (95% CI)**			**0**			**0**		**Not estimable**
Heterogeneity: Not applicable								
Test for overall effect: Not applicable								

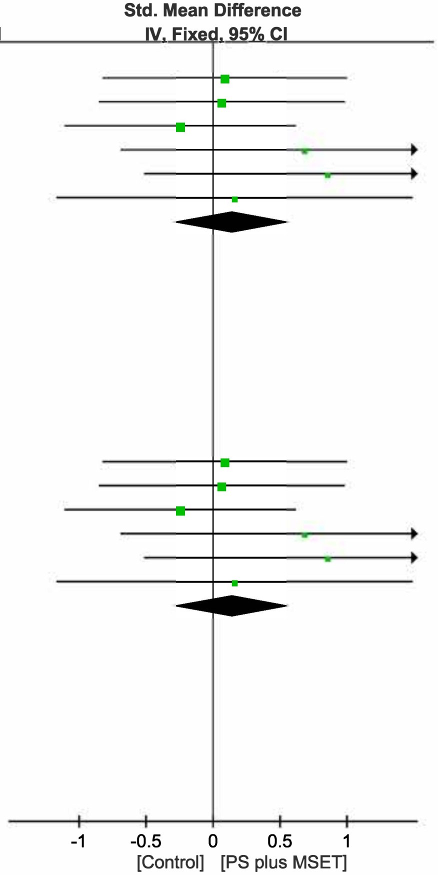

Figure S10. Forest plot summarizing effects of protein supplement (PS) plus muscle strength exercise training (MSE) on single leg stance at an overall duration and each follow-up time point. The horizontal line links the lower and upper limits of the 95% CI of this effect. The combined effects are plotted using black diamonds. 95% CI = 95% confidence interval; Std. = standard; IV = inverse variance; CG = control group; Con = control; MET = multicomponent exercise training; PLA-S, placebo supplement; RET = resistance exercise training.
